# DeepFlower: a deep learning-based approach to characterize flowering patterns of cotton plants in the field

**DOI:** 10.1186/s13007-020-00698-y

**Published:** 2020-12-07

**Authors:** Yu Jiang, Changying Li, Rui Xu, Shangpeng Sun, Jon S. Robertson, Andrew H. Paterson

**Affiliations:** 1 Horticulture Section, School of Integrative Plant Science, Cornell AgriTech, Cornell University, Geneva, NY 14456 USA; 2 Phenomics and Plant Robotics Center/College of Engineering, The University of Georgia, Athens, GA 30602 USA; 3 College of Agricultural & Environmental Sciences, The University of Georgia, Athens, GA 30602 USA; 4 Franklin College of Arts and Sciences, The University of Georgia, Athens, GA 30602 USA

**Keywords:** Flowering pattern, Deep learning, Object detection, High-throughput plant phenotyping, Image analysis.

## Abstract

**Background:**

Flowering is one of the most important processes for flowering plants such as cotton, reflecting the transition from vegetative to reproductive growth and is of central importance to crop yield and adaptability. Conventionally, categorical scoring systems have been widely used to study flowering patterns, which are laborious and subjective to apply. The goal of this study was to develop a deep learning-based approach to characterize flowering patterns for cotton plants that flower progressively over several weeks, with flowers distributed across much of the plant.

**Results:**

A ground mobile system (GPhenoVision) was modified with a multi-view color imaging module, to acquire images of a plant from four viewing angles at a time. A total of 116 plants from 23 genotypes were imaged during an approximately 2-month period with an average scanning interval of 2–3 days, yielding a dataset containing 8666 images. A subset (475) of the images were randomly selected and manually annotated to form datasets for training and selecting the best object detection model. With the best model, a deep learning-based approach (DeepFlower) was developed to detect and count individual emerging blooms for a plant on a given date. The DeepFlower was used to process all images to obtain bloom counts for individual plants over the flowering period, using the resulting counts to derive flowering curves (and thus flowering characteristics). Regression analyses showed that the DeepFlower method could accurately (R^2^ = 0.88 and RMSE = 0.79) detect and count emerging blooms on cotton plants, and statistical analyses showed that imaging-derived flowering characteristics had similar effectiveness as manual assessment for identifying differences among genetic categories or genotypes.

**Conclusions:**

The developed approach could thus be an effective and efficient tool to characterize flowering patterns for flowering plants (such as cotton) with complex canopy architecture.

## Background

Flowering is one of the most important processes for angiosperms (flowering plants), reflecting the transition from vegetative to reproductive growth and significantly affecting crop yield and adaptability to various environments. Therefore, characterization of flowering patterns would not only facilitate studies for understanding angiosperms genetically and physiologically, but also holding potential to contribute to breeding of new cultivars for optimal yield and environmental adaptability [1–3].

Conventionally, studies related to plant flowering patterns have required human evaluators to check experiment fields and record flowering status manually. For instance, plants and plots can be checked regularly by human evaluators to monitor characteristics such as the number of days after planting (DAPs) to the first bloom. In addition, human evaluators often used a categorical scoring system to assess flowering stages (e.g., estimating when 10% of plants in a plot have opened blooms), so that the time duration between particular flowering stages can be calculated. Human recorded flowering data have helped researchers to study flowering patterns for several important crops such as maize [4], rice [5], cereal [6], and sorghum [7]. Human evaluation, however, has two major disadvantages. First, the evaluation is subjective, which means that different human evaluators might give different scores to an individual plant/plot. As a result, human-evaluated flowering data could contain a substantial amount of noise. Second, human evaluation is laborious, and presents great challenges for large-scale experiments and breeding programs. An automated high throughput approach to characterize flowering patterns can mitigate each of these disadvantages.

Advances in high throughput plant phenotyping (HTP) and breakthroughs in deep learning enable the possibility of rapid characterization of flowering patterns for plants in the field. Several studies demonstrated the use of deep convolutional neural networks (CNNs) and meta-models (e.g., Faster RCNN developed by Ren et al. [8]) to detect and count fruit in images for crops such as mangoes [9], apples [10], and sweet peppers [11]. Although these studies achieved relatively high counting accuracies (R^2^ > 0.92), they were primarily used for “one time” measurements of yield estimation. A big challenge to flowering characterization is that in many plants it occurs over a long period of time, requiring one to frequently detect and count newly opened blooms on plants.

Recent studies have intensively investigated deep learning-based solutions to flower detection and counting for field crops such as wheat [12–16], corn [17], sorghum [18], rice [19], and cotton [20]. Based on the counting strategies, these methods can be classified into three categories: regression-based, classification-based, and detection-based counting [21]. Regression-based counting is a one-stage strategy and extracts features using CNNs to directly regress a continuous count of flowers/floral structures in images. Classification-based counting is a similar strategy but it classifies images into a class representing a discrete count/percentage of flowers/floral structures. The two counting strategies considerably reduce the training complexity and the cost of data annotation. Experiments showed that they can provide fairly good counting accuracies (up to 90%) [13, 15–17]. However, the regression- and classification-based methods may suffer from overfitting problems because the CNN models are generally much more complex than training objectives (a numeric value or several classes). Careful designing of network architecture would be necessary for good performance and generalizability. In addition, the spatial information of flowers/floral structures cannot be obtained from regression/classification results, which limits the potential for flower distribution analysis and actuation-based applications such as flower thinning in crop load management. To address or mitigate these issues, the detection-based counting strategy has been used in very recent studies [12, 18–20]. The detection-based counting is a two-stage strategy that detects flowers/floral structures in images and then counts the number of detections. A study reported that the detection-based counting could provide better counting accuracy and robustness than regression-based ones, showing the greatness of the detection-based strategy for flower counting over a growing season [12]. Among many studies for flower detection and counting in the past two years, only three of them used the developed counting methods to monitor plant flowering during the entire flowering period to characterize key flowering patterns such as heading date [13, 15, 19]. All three studies demonstrated high accuracies of heading date estimation (up to an error of 2 days) because of superior performance of deep learning models for flower detection and counting. On the other hand, having only few studies capable of monitoring plant flowering reiterates the challenges of intensive data collection and analysis over an extended period for the characterization of flowering patterns.

It is also noteworthy that nearly all studies focused on the crops with simple shoot architecture where flowers/floral structures form on top of plant canopies, which significantly simplifies data collection and flower detection and counting. In contrast, cotton plants have much more complex shoot architecture and can form flowers across the entire plant, presenting extreme challenges to the detection and counting of cotton flowers. To date, only one study reported a two-stage approach to detect and count blooms (white flowers) in cotton plots from aerial images. The approach first segmented candidate regions of blooms using a thresholding method, and subsequently classified the candidate regions as bloom or non-bloom using a custom CNN to count the number of blooms in individual cotton plots. Although the two-stage approach showed some success in counting blooms, it had a major limitation in that a considerable portion of blooms could not be captured by aerial images because of occlusions, resulting in a relatively large underestimation of bloom counts. In addition, this study only measured bloom counts for several days, lacking the capability for flowering pattern analyses over a long flowering period. To overcome the aforementioned limitations, we need improvements and modifications in both technical and agronomic aspects. For the technical aspect, it is necessary to explore the use of side-view proximal imaging and CNN-based object detection to detect and count individual blooms over an entire flowering period. For the agronomic aspect, the planting scheme needs to be modified from the conventional plot-based layout to a single-plant layout (SPL) where individual plants are treated as plots with a wide row spacing of 1.52 m. In such a planting scheme, flower occlusions due to dense plant canopies can be minimized to increase the flower visibility and therefore detection and counting accuracies. Based on our previous studies [22], SPL-based experiments could provide phenotypic data revealing significant differences among genotypic categories or genotypes, showing the great potential of studying flowering patterns for cotton in the field.

The goal of our study was to develop a deep learning-based approach to characterize flowering patterns of cotton plants in the field. Specific objectives were to (1) develop a multi-view imaging system that can acquire cotton plant images in a high throughput manner; (2) develop a deep learning-based approach (DeepFlower) to detect and count emerging blooms and to characterize flowering patterns for individual plants; and (3) evaluate the accuracy and efficacy of the developed approach for the identification of differences in flowering patterns among genetic categories and genotypes.

## Results

### Representative detection results

**Fig. 1:**
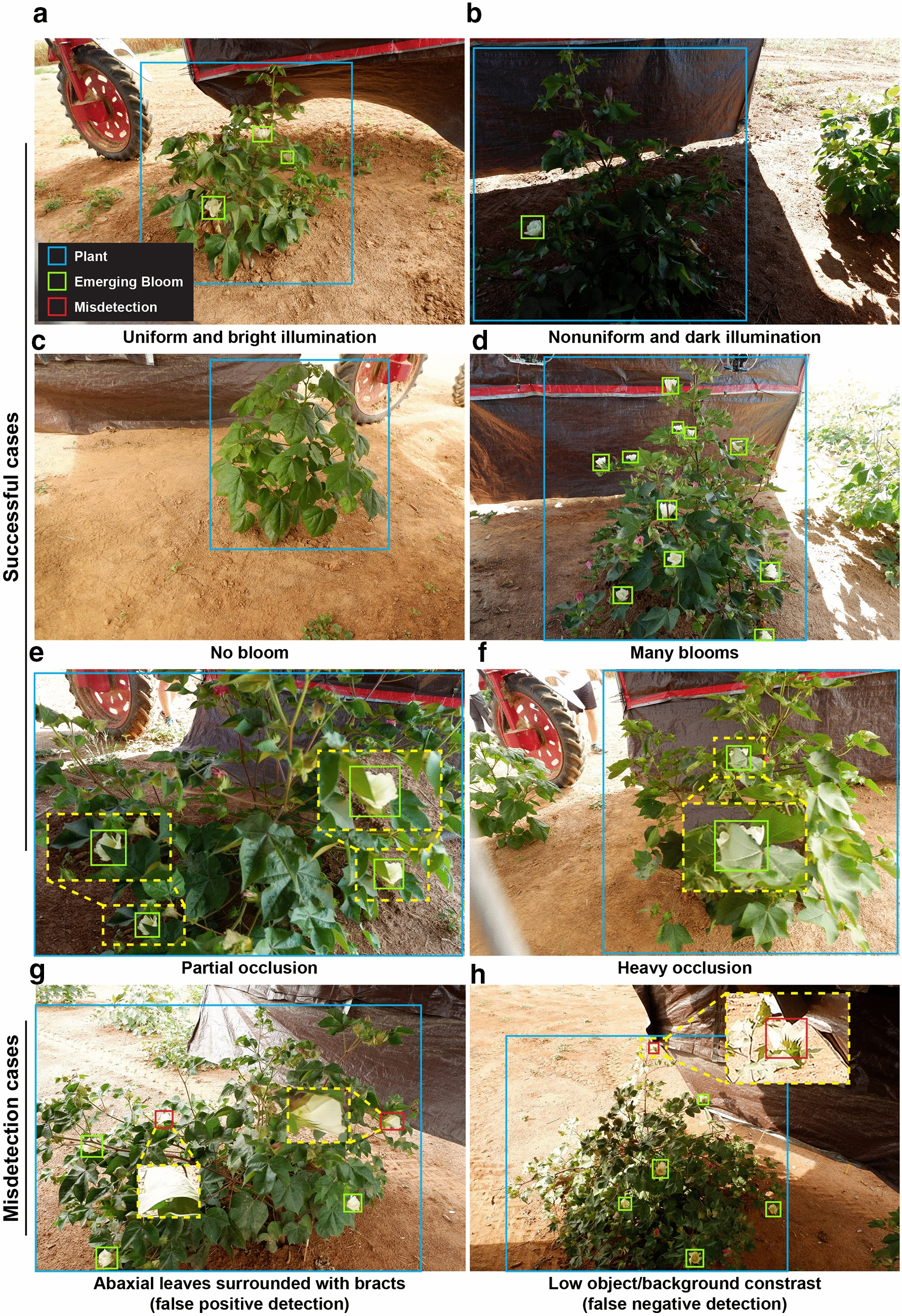
Representative results of plants and emerging blooms detected by the trained Faster RCNN (FrRCNN5-cls) model. The top three rows demonstrate successful detections under different illumination, bloom load, and occlusion conditions. The bottom row shows two failed cases of emerging bloom detection, one because back-sided leaves had higher reflectance and were identified incorrectly as emerging blooms, and the other because a lower contrast between emerging blooms and the background could lead to mis-detection

Generally, the Faster RCNN model (FrRCNN_5-cls_) could accurately detect plants and emerging blooms under different illumination, bloom load, and occlusion conditions (Fig. 1). If a proper viewing angle was used, the enclosure mostly provided uniform and bright illumination (e.g., Fig. 1a). The illumination could be an issue as the enclosure did not fully cover the imaging area. When the solar zenith angle was steep or the camera was configured to face the enclosure entrance, the field of view (FOV) of cameras could include both shaded and strongly illuminated areas. Consequently, collected images could have very dark illumination for the shaded part, making it difficult to identify objects with low reflectance (e.g., the plant in Fig. 1b). The FrRCNN_5-cls_ model learned feature representations to detect the plant, showing its capability to handle object variations because of extreme illumination changes. In addition to illumination, bloom load also varied dramatically during the entire flowering process. Plants would have fewer emerging blooms in early and late stages than the peak flowering time. The FrRCNN_5-cls_ model provided accurate detection results for both cases, showing the efficacy of using a single model to process images of plants in different flowering stages. Occlusion was another great challenge for detecting emerging blooms. Cotton plants were branchy and leafy during the flowering period, so blooms were frequently occluded by plant leaves and branches. Depending on the cultivar and development stages, the occlusions varied in direction and severity (see Fig. 1e, f), which introduced issues for object detection (especially by using traditional image processing). The FrRCNN_5-cls_ model learned effective features to describe and detect occluded emerging blooms, especially some heavily occluded emerging blooms (Fig. 1f). All of these successful cases demonstrated the capability of the FrRCNN_5-cls_ model to detect plants and emerging blooms in images with varied conditions.

The FrRCNN5-cls model, however, could not process certain cases. The abaxial surface of leaves has a higher reflectance than the adaxial surface (Cordon and Lagorio, 2007), showing a similar contrast pattern with emerging blooms (brighter than adjacent areas). When the abaxial surface of leaves was exposed to the camera and surrounded with bracts, these leaves could not be differentiated easily from true emerging blooms by even human observation (Fig. 1g), thereby generating false positive detections of emerging blooms. In addition, because of a high reflectance, emerging blooms under strong illumination lost the contrast with background and detailed textures, and thus became considerably more difficult to be identified in the images. In this situation, emerging bloom objects were not accurately detected by the FrRCNN5-cls model.

### Results of ablation experiments

#### Labeling strategy

Two labeling strategies were used in this study: 3-class and 5-class labeling strategies. The 3-class labeling strategy included the classes of target plant, emerging bloom, and non-bloom objects, whereas the 5-class labeling strategy further split the non-bloom class into three classes, resulting in five classes of target plant, emerging bloom, region with specular reflectance, opened boll, and others.

**Fig. 2:**
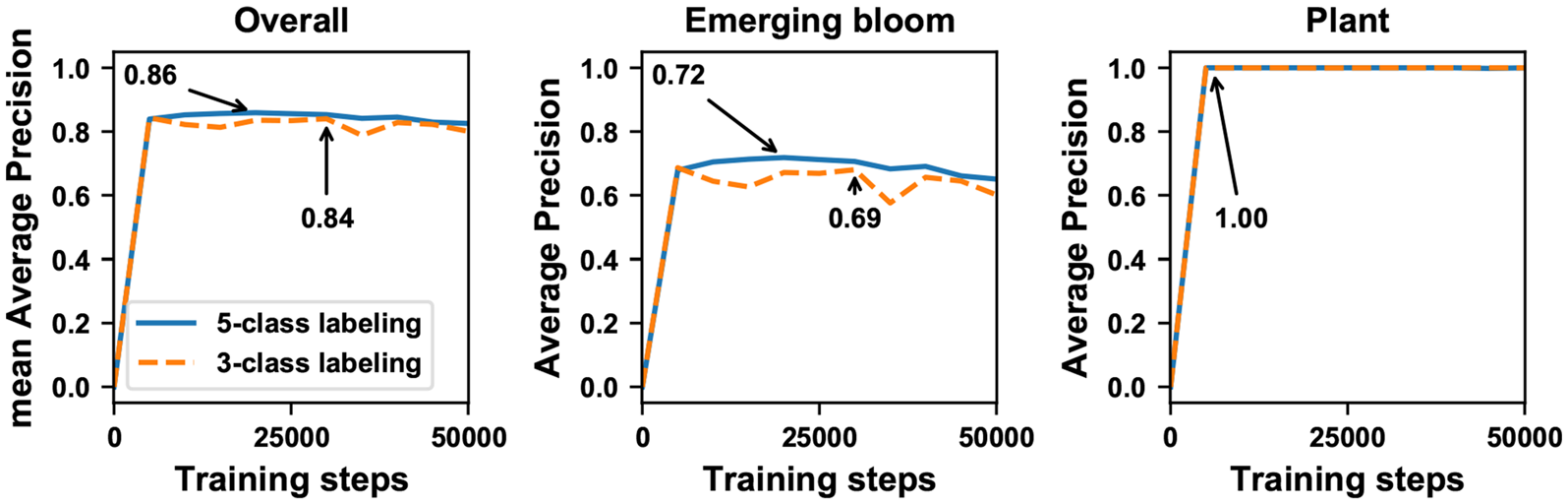
Detection accuracies (mean average precision and average precision per class) on the validation dataset by using models trained with datasets labeled by the 5-class (FrRCNN_5-cls_) and 3-class (FrRCNN_3-cls_) methods, respectively. The mean average precision (mAP) was calculated over the bloom and plant classes only

Overall, the model (FrRCNN_5-cls_) trained using the 5-class labeling strategy had improved performance (mean average precision, mAP and average precision per class, AP) than that (FrRCNN_3-cls_) trained using the 3-class labeling strategy (Fig. 2). In particular, the AP of emerging bloom detection increased by 3% by using the 5-class labeling strategy. Compared with 3-class labeling, the 5-class labeling strategy could more efficiently split classes with similar appearance. Consequently, the variation within a class would become smaller than the differences between classes, providing benefits for training deep neural networks. For instance, there could be several types of non-bloom objects that had a distinct appearance. There were also some bright gaps between plant branches and leaves, which formed regions that had a similar appearance to emerging blooms, whereas there were some other objects (e.g., camera) that looked dissimilar from emerging blooms (see “specular reflectance” and “others” objects in Additional file 1: Figure S1). If these regions/objects were labeled with different classes, it would be relatively easier for deep neural networks to learn features to form classification boundaries for separating classes with similar appearance. Otherwise, deep neural networks might not learn effective features, resulting in misclassification between regions/objects with similar appearance.

It is noteworthy that the AP score of the bloom class was 0.72 even by using the 5-class labelling strategy, meaning that models could detect irrelevant regions as blooms and lead to the over-detection issue. Thus, by using a high classification confidence score (0.7 in this study), we expect to mitigate this issue and provide accurate detection results for bloom counting.

#### Counting strategy

**Fig. 3:**
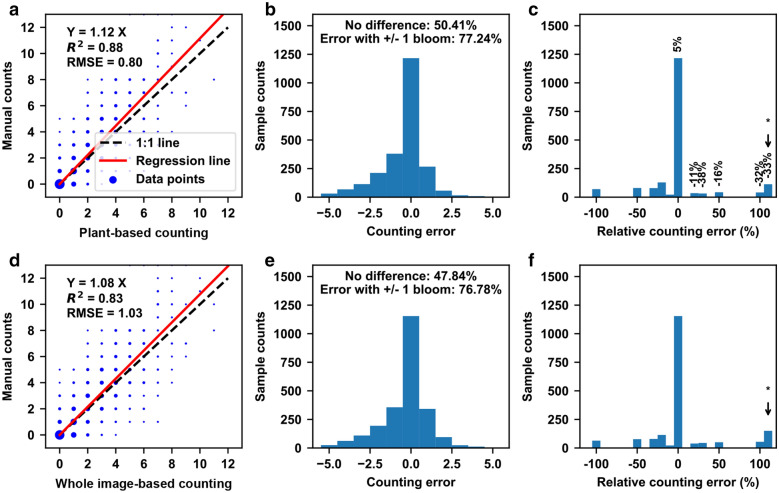
Counting accuracies calculated using “plant-based counting” (top row) and “whole image-based counting” (bottom row) strategies, respectively, for individual plants on each of the 26 scanning dates (a total of 2834 data points). **a** and **d** are linear regression results between the imaging derived and manual counts. **b** and **e** are the histograms of counting errors. C and F are the histograms of relative counting errors for samples with an absolute counting error of less than 1. In **c** and **f**, the numbers on top of the bars indicate the relative improvement (over 5%) of using the “plant-based counting” strategy over the “whole image-based counting” strategy. The asterisk denotes samples that had a zero count with the manual method but a non-zero count with the imaging method

Overall, for each image, the “plant-based counting” strategy provided improved accuracy over the “whole image-based counting” strategy (Fig. 3). Although the regression slope calculated using the “plant-based counting” strategy was slightly higher than that calculated using the “whole image-based counting” strategy, a higher correlation and lower root mean squared errors (RMSE) were achieved by using the “plant-based counting” strategy, indicating improved counting accuracy (Fig. 3a and d). These improvements were primarily because the “plant-based counting” strategy made more samples in the counting error range within ± 1, especially a 3% increase with no counting difference (Fig. 3b and e). As an absolute counting error of one bloom might be substantial when plants had very few emerging blooms (e.g., early and late flowering stages), relative counting errors were calculated for samples with counting errors less than one bloom (Fig. 3c and f). Compared with the “whole image-based counting” strategy, the “plant-based counting” strategy increased the number of samples with no relative counting error by 5% and dramatically reduced the number of samples with relative counting errors over 20%. It is also noteworthy that the “plant-based counting” strategy dramatically improved the counting accuracy for samples that had a zero count with the manual method but a non-zero count with the imaging method (denoted by asterisks in Fig. 3c and f).

**Fig. 4:**
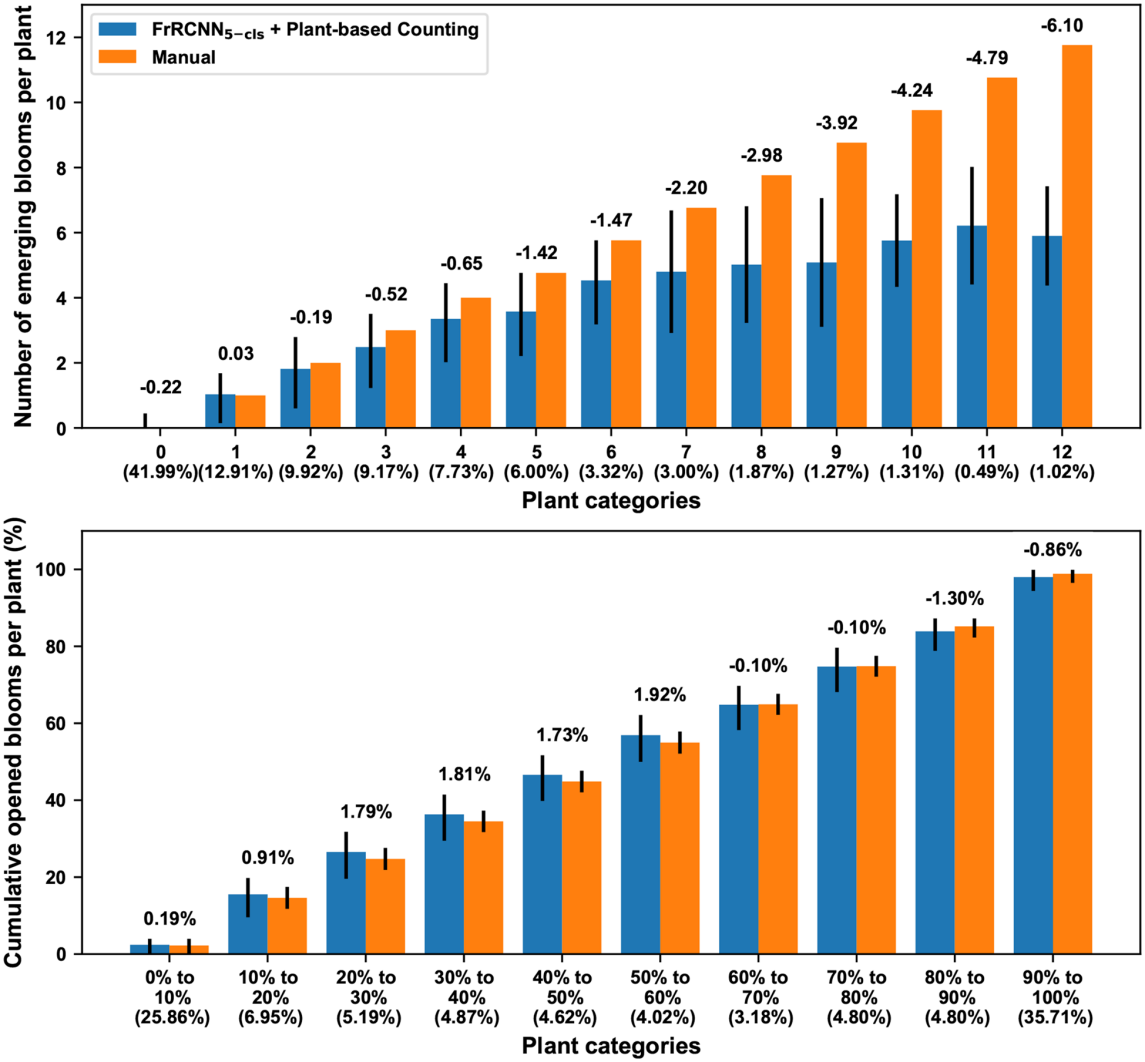
Errors of absolute counting (top chart) and cumulative percentage (bottom chart) for emerging blooms per plant by using the FrRCNN_5-cls_ and the “plant-based counting” strategy. For absolute counting, plants were grouped into 13 categories based on the number of emerging blooms (0 to 12) on those plants on a particular date. For cumulative percentage, plants were grouped into 10 categories (from 0–10% to 90–100%) of opened blooms on those plants on a particular date. The number on top of each grouped bar indicated the difference between counts (or cumulative percentage) calculated using the imaging and manual methods

Although the FrRCNN_5-cls_ and the “plant-based counting” strategy demonstrated improved performance on emerging bloom detection and counting, respectively, significant counting errors were identified by jointly using the FrRCNN_5-cls_ and the “plant-based counting” strategy as a counting approach (Fig. 4). For absolute counting, the combination of the FrRCNN_5-cls_ model and the “plant-based counting” strategy provided accurate measurements (less than one bloom) for plants with zero to four emerging blooms per day (approximately 82% of cases). Absolute counting errors substantially increased, however, when plants had five or more emerging blooms (approximately 18% of cases). On average, the developed counting approach also reached a plateau of 6 blooms per plant per day. Thus, when plants reached peak flowering time (over 10 emerging blooms per day), absolute counting errors were over 4 blooms per plant per day, which was equivalent to about 50% relative counting errors. This occurred primarily because of the assumption in the developed counting approach that a single image from a particular viewing angle would capture most (or even all) emerging blooms on a plant on one day, and thus the counting approach could obtain the maximum bloom count from one out of four images for a single plant. This assumption generally held true in flowering stages when plants had a small number of emerging blooms per day, so the counting approach provided accurate counts for most plants. This assumption, however, was invalid during the peak flowering time when plants had a large number of emerging blooms per day. Furthermore, emerging blooms were distributed around plant canopies, so a single image from any viewing angle would not be sufficient to capture all blooms on a plant, resulting in significant underestimation of absolute bloom counts.

The significant underestimation of absolute bloom counts, however, showed a limited influence on the accuracy of calculating cumulative percentages of opened blooms. Errors in the cumulative percentage of emerging blooms were less than 2% irrespective of flowering stages. There were two possible reasons. First, images with significant underestimation occupied only a small portion (~ 2.72%) of the entire dataset, meaning that on average, the large underestimation only affected individual plants on few days. Thus, a limited influence was observed on cumulative percentage of opened blooms. Second, cumulative percentage was the ratio of total opened blooms from the beginning of flowering to a specific day and total opened blooms over the flowering period. The underestimation of absolute counts would be included in both the numerator and denominator of the cumulative percentage, and thus mitigated somewhat. This would be particularly true if a genotype could intensively produce blooms (more than 10 blooms daily) in a short time of period. In such a case, a relatively similar scaling factor was introduced to both the numerator and denominator of the cumulative percentage, which largely reduced the underestimation effect. Therefore, the use of cumulative percentage could reasonably address a concern in that the developed method might have different accuracies for genotypes with different flowering patterns. Nonetheless, the high accuracy of the calculated cumulative percentage of opened blooms could hold great potential for flowering characterization.

### Results of flowering characteristics and statistical analyses

#### Representative flowering curves

As the developed counting approach underestimated the number of emerging blooms on plants during the peak flowering time, the absolute bloom counting curves generated using imaging-derived counts also showed large differences from those generated using manual counts during that time (Additional file 1: Figure S2). This suggests that the curves should not be used for quantitative analyses such as the maximal number of emerging blooms per plant over a growing season. The flowering curves derived by the imaging method, however, generally showed a similar trend as the curves derived by the manual method, suggesting their utility for certain qualitative analyses. For instance, the field received precipitation (approximately 8 mm of rain) and experienced chilling temperatures (approximately 10 °C cooler than the monthly-average temperature) on 16 September 2018 (95 DAPs) and 24 September 2018 (103 DAPs), respectively. After the weather changed, the plants mostly had a reduced number of emerging blooms on the next sampling day in flowering curves derived by both the manual and imaging methods. Certain genotypes (e.g. Exotic T0368BC3MDN GH196 and Elite DES 56), however, did not show such a pattern, perhaps indicating that they are more resistant to severe weather changes than other genotypes. (Additional file 1: Figure S3).

**Fig. 5:**
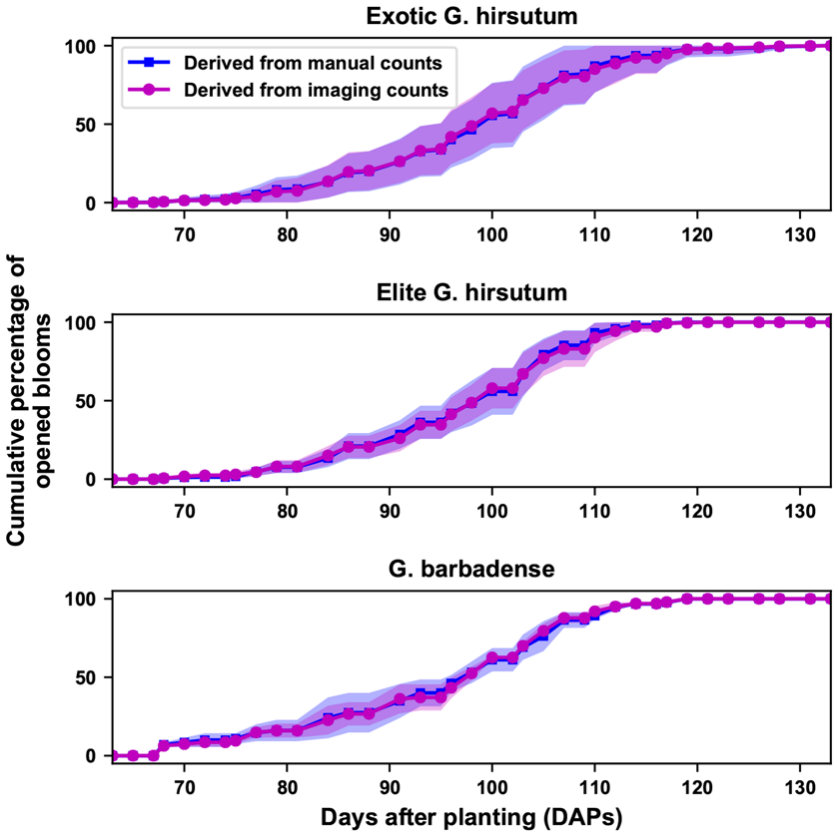
Cumulative flowering curves derived using imaging and manual counts for three genetic categories (elite *G. hirsutum*, exotic *G. hirsutum*, and *G. barbadense*). Group mean values are drawn in lines (solid and dashed lines for results derived by the imaging and manual methods, respectively), and group standard deviations are indicated by shaded areas (magenta and blue for results derived by the imaging and manual methods, respectively)

Cumulative flowering curves generated using bloom counts derived by the imaging method were very similar to those generated using manual counts (Fig. 5). The same correspondence was also observed for individual genotypes (Additional file 1: Figure S4). This suggests that the curves derived using the imaging method could potentially be used for both qualitative and quantitative characterization of flowering patterns. Two distinctive patterns were observed from the curves. First, exotic *G. hirsutum* presented larger within-group variation than elite *G. hirsutum* and *G. barbadense*. This was because the exotic group contained wild genotypes that are diverse in their flowering patterns, whereas elite *G. hirsutum* have been selected for flowering patterns that conferred optimal yield. There was only one cultivar in the *G. barbadense* group, which should not present large variation. Second, both exotic and elite *G. hirsutum* showed a relatively steeper slope than *G. barbadense*, indicating a potential difference in flowering duration between various species.

#### Statistical analysis results

Significant interaction effects were presented on extracted flowering characteristics (first bloom date, flowering start date, and flowering duration) between the genotype and transplanting date, suggesting the necessity of analyzing flowering patterns for each transplanting batch separately (see Additional file 1: Tables S1–S6 for detailed ANOVA analysis tables). As only the much larger first transplanting batch showed statistical significance among genetic categories or genotypes, successive sections focused on data of the first transplanting batch.

**Fig. 6:**
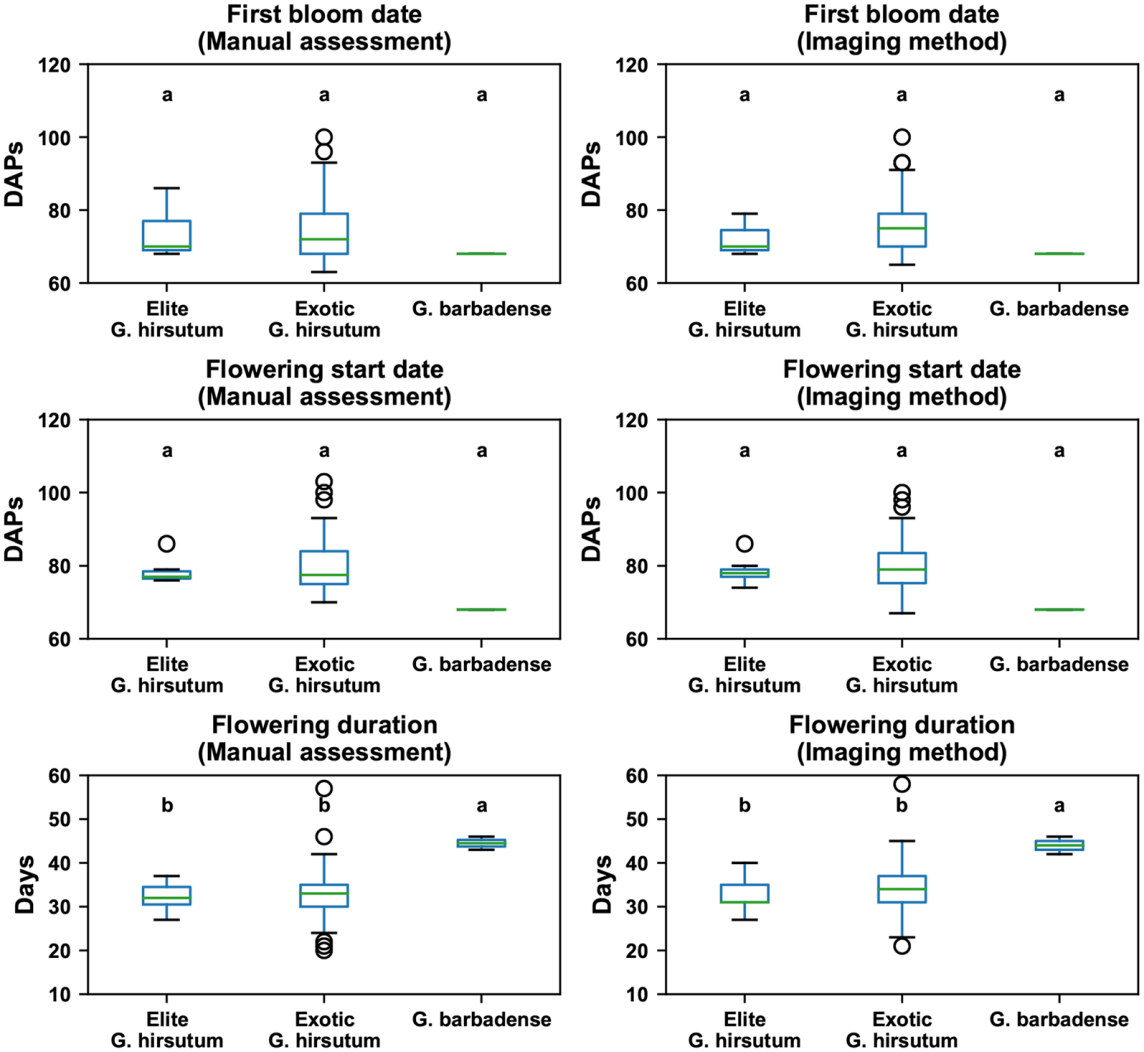
Boxplot of flowering characteristics (first bloom date, flowering start date, and flowering duration) among three genetic categories (elite *G. hirsutum*, exotic *G. hirsutum*, and *G. barbadense*) in the first transplanting batch. Groups with a statistically significant difference (*p* < 0.05) are denoted with different letters, and group mean values of each characteristic are sorted alphabetically

Flowering characteristics calculated using the flowering curves derived by the imaging method showed the same statistical power in differentiating the three genetic categories as those calculated using the flowering curves derived by the manual method (Fig. 6). For the first bloom date and flowering start date, although *G. barbadense* showed the lowest values with the least standard deviation, it could not be statistically separated from the *G. hirsutum* groups for two reasons. First, exotic *G. hirsutum* contained diverse genotypes, presenting large variation that covered the other two groups. Second, *G. barbadense* had only two replicates in the first transplanting batch, which had limited statistical power to be differentiated from other groups. For flowering duration, however, *G. barbadense* was significantly longer than the *G. hirsutum* groups, which was an expected flowering pattern for *G. barbadense* (Pima cotton) in the study area.

**Fig. 7:**
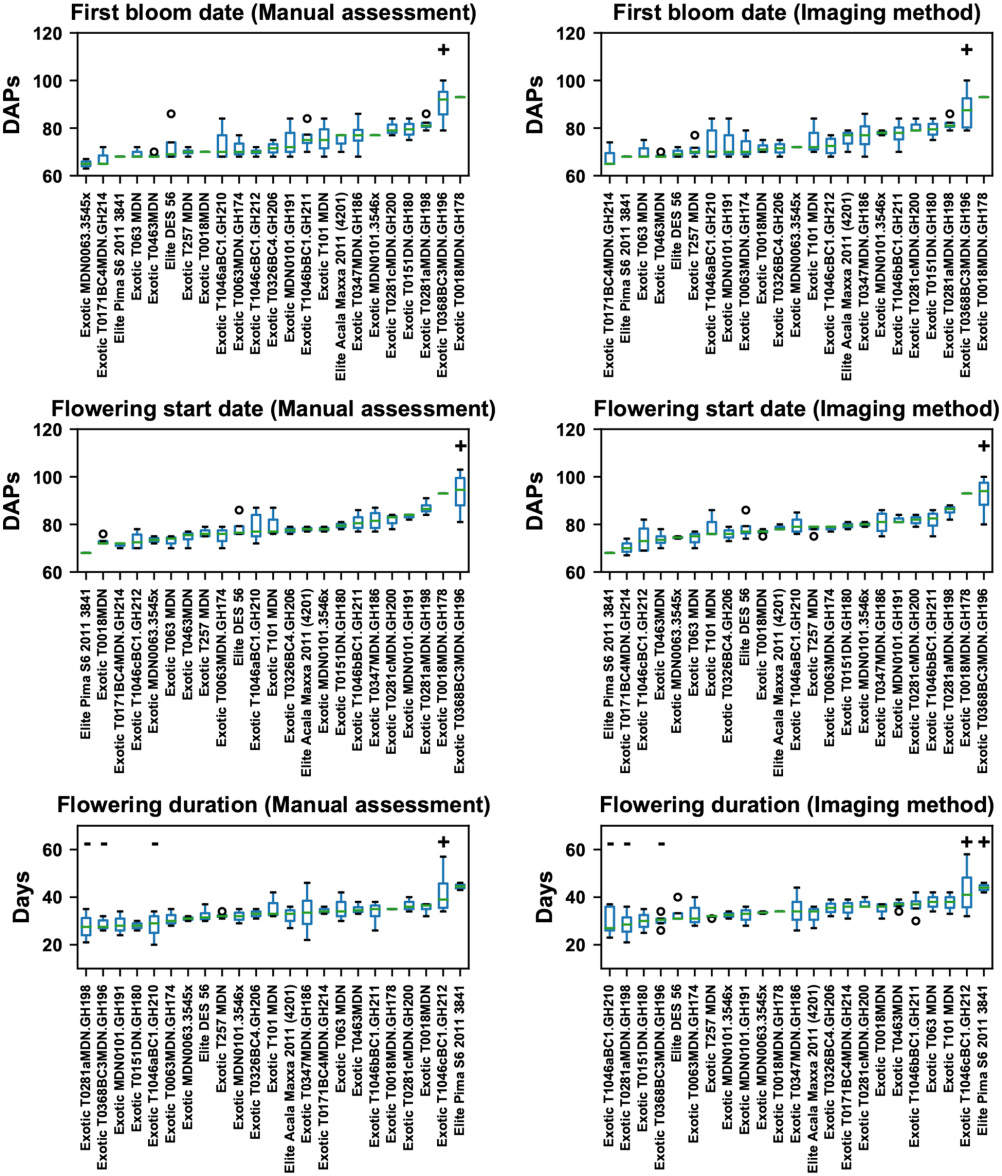
Boxplot of flowering characteristics (first bloom date, flowering start date, and flowering duration) among 23 genotypes in the first transplanting batch. Genotypes with statistically higher values are denoted by “ + ”, whereas genotypes with statistically lower values are denoted by “−”. Differences were inferred at the significance level of 0.05

While the order of individual genotypes was slightly different, flowering characteristics derived by the imaging and manual methods showed very similar statistical patterns among genotypes (Fig. 7). Genotype T0368BC3MDN.GH196 had the first bloom (first bloom date) and entered into the flowering period (flowering start date) significantly later than other genotypes, suggesting that it could be used for studying genes and gene regions controlling flowering time. In addition, genotype T0368BC3MDN.GH196 had a significantly shorter flowering duration than other genotypes. This occurred likely because of environmental effects. Overall, air temperature decreased dramatically (more than 15 °C) after 120 DAPs, leading to a sudden drop of emerging blooms. Although several blooms opened after 120 DAPs (see Additional file 1: Figure S3), the freezing temperature might cause an early termination of flowering for T0368BC3MDN.GH196. Some other genotypes also presented significant differences in flowering duration, such as genotype T1046cBC1.GH212 for a longer duration and genotypes T0281aMDN.GH198 and T1046aBC1.GH210 for a shorter duration. It should be noted that genotype Pima.S6.2011.3841 had a statistically longer flowering duration using the characteristic derived from manual counts but not by that derived from imaging counts, which was the only difference in the statistical patterns between the two methods. This possibly occurred because manual counts would not miss any emerging blooms on a plant, having a relatively stronger capability to identify differences between genotypes with fewer replicates.

Based on the estimation, each genotype should have at least 2 replicates to ensure adequate statistical power to identify the significance of the first bloom date and flowering start date, or at least 3 replicates to ensure the statistical power to identify the significance of flowering duration among the 23 genotypes (Table 1). As there were only 2 replicates per genotype Pima.S6.2011.3841, no significant difference in flowering duration was identified between Pima.S6.2011.3841 and other genotypes, which agreed with the experimental result. If the variation because of genotype remains the same as that in the first transplanting batch, using more genotypes (e.g., 200 genotypes in a population) would slightly increase the statistical power for identifying significance among genotypes. Flowering curves derived by the imaging method, therefore, would remain effective for flowering pattern analyses in large-scale experiments.

**Table 1:** Estimated number of replications per genotype at the significance level of 0.05 and power of 0.95

Trait	Batch	Effect size F	Number of genotypes	Estimated number of replications
FBD	1	1.18	22	2
FBD*	1	1.18	200	2
FSD	1	1.52	22	2
FSD*	1	1.52	200	2
FD	1	0.88	22	3
FD*	1	0.88	200	2

FBD shorts for first bloom date, FSD shorts for flowering start date, and FD shorts for flowering duration. The asterisk denotes the estimation for one population in a NAM study

### Data collection frequency

Average differences between mean cumulative flowering curves derived by the imaging and manual methods increased when data collection frequency was reduced (Table 2). Generally, the increase of average curve difference was inversely proportional to the reduction of data collection frequency. This suggests that the temporal resolution of data (determined by data collection frequency) is also an important factor for the accuracy of cumulative flowering curves. With a higher temporal resolution, daily bloom counts could be obtained more frequently, which could improve the accuracy of calculating the total number of opened blooms and cumulative percentages of opened blooms over a flowering period.

**Table 2:** Average differences between cumulative flowering curves derived by the imaging and manual methods

Data collection frequency	Exotic *G. hirsutum*	Elite *G. hirsutum*	*G. barbadense*
Once per week (10 dates)	10.96%	9.92%	12.01%
Twice per week (20 dates)	3.79%	3.85%	4.23%
Three times per week (26 dates)	1.03%	1.21%	1.27%

Statistical results showed that flowering characteristics extracted from the subset with the frequency of twice per week had the same statistical power in identification of genotypic groups as those extracted from the original dataset (see Additional file 1: Figure S5). The statistical power, however, was lost when the data collection frequency was further reduced to once per week (compare flowering duration in Additional file 1: Figure S6 and Fig. 6). This suggests that an optimal data collection frequency would be twice per week for the current study, which can provide adequate statistical power in genotype differentiation and dramatically reduce the workload of field data collection.

## Discussion

The DeepFlower approach demonstrated the efficacy of detecting and counting emerging blooms in images to characterize flowering patterns for different genetic categories or genotypes. Flower characteristics derived by the imaging method showed an almost identical capability for identifying significance among genotypes with manual counts, which further validated the effectiveness of the DeepFlower approach for studying flowering patterns. In particular, the DeepFlower approach successfully revealed flowering patterns for cotton plants that have a complex canopy architecture (and thus difficulties of emerging bloom detection and counting) and therefore should transfer well to other flowering plants that have the same or similar canopy architecture. This suggests that the combination of the image acquisition system and DeepFlower approach can be an effective and efficient tool for characterization of flowering patterns for plants in the field, holding great potential for identifying gene loci that control flowering behavior for different plant genotypes.

Although the DeepFlower approach showed promising results for extracting flowering characteristics, several aspects can be further improved or explored. First, the scanning throughput is relatively low for the current configuration. The platform ran at approximately 0.25 m/s and took around 25 min to complete the scanning of the present experimental field (approximately 0.05 ha), resulting in a scanning throughput of 0.12 ha/h. This throughput might not be adequate for extremely large experiments, e.g., that involves up to several thousand genotypes with at least two replicates per genotype (up to a couple of hectares). Challenges, however, would need to be identified to balance the platform cost (camera with high resolution and fast frame rate), image quality (blurry), and scanning throughput (platform moving speed). Second, the present DeepFlower approach oversimplifies the counting task by using only one single image with the maximum count among the four viewing images. The approach depends upon the assumption that most or all emerging blooms can be seen from a single one of these four viewing angles. Experimental results, however, showed that this assumption is invalid when plants enter into peak flowering time, leading to a significant underestimation of bloom counts. Consequently, absolute bloom counting curves cannot be used for quantitative analysis of flowering patterns. A viable solution is to integrate 3D imaging so that 2D detections can be projected onto a global 3D space for counting. For instance, photogrammetric methods (e.g., structure from motion) can be used to reconstruct 3D point clouds using images from multiple viewing angles, so that for a single plant emerging bloom detections can be projected from different 2D images onto a global 3D space to remove duplicated detections (and thus counts). In the present study, preliminary tests using the collected images suggested that images from four viewing angles (approximately 90° apart from each neighboring angle) could not provide adequate image overlap to reconstruct 3D point clouds of a single plant, and thus the 2D to 3D projection. It is therefore necessary to conduct successive studies to explore the optimal image collection configuration (e.g., viewing angles and number of images) for 3D reconstruction using photogrammetric methods. Another way is to fuse 2D images with 3D point clouds acquired using separate instruments (e.g., LiDARs), which enables the 2D to 3D projection. This will also require considerable efforts to develop new sensing systems for data collection and algorithms for data fusion (especially multi-source heterogeneous data fusion). Last, although the DeepFlower approach demonstrated great performance for the SPL-based experiment, it could not fully address the flower counting problem in plot-based layouts (either single- or double-plot per row) that have been widely used in cotton research and production. This could raise particular concerns on transferring findings and knowledge from the SPL-based experiments to practical production systems. Thus, in the future, it would be necessary to further explore the possibility of combining engineering, agronomy, genetics/genomics, and statistics approaches for an interdisciplinary solution that can fully address the flower counting problem in an environment closer to practical situations.

## Conclusions

The developed imaging approach (combination of the image acquisition system and DeepFlower approach) can be an efficient and effective tool for detecting and counting blooms on plants in the field, demonstrating promising results for the characterization of flowering patterns. In particular, the developed approach can potentially be used for many other flowering plants that have a simpler or similar canopy architecture, providing potential for deepening the understanding of the flowering process in general. Future studies will be focused on the integration of 3D imaging to further improve the counting accuracy and expand the capability of mapping bloom positions on plants. Moreover, it is needed to incorporate the developed approach with advanced statistics methods and experimental designs for the cotton flower counting in conditions closer to practical scenarios such as plot-based layouts.

## Materials and methods

### Image acquisition

#### High throughput imaging system and experimental design

**Fig. 8:**
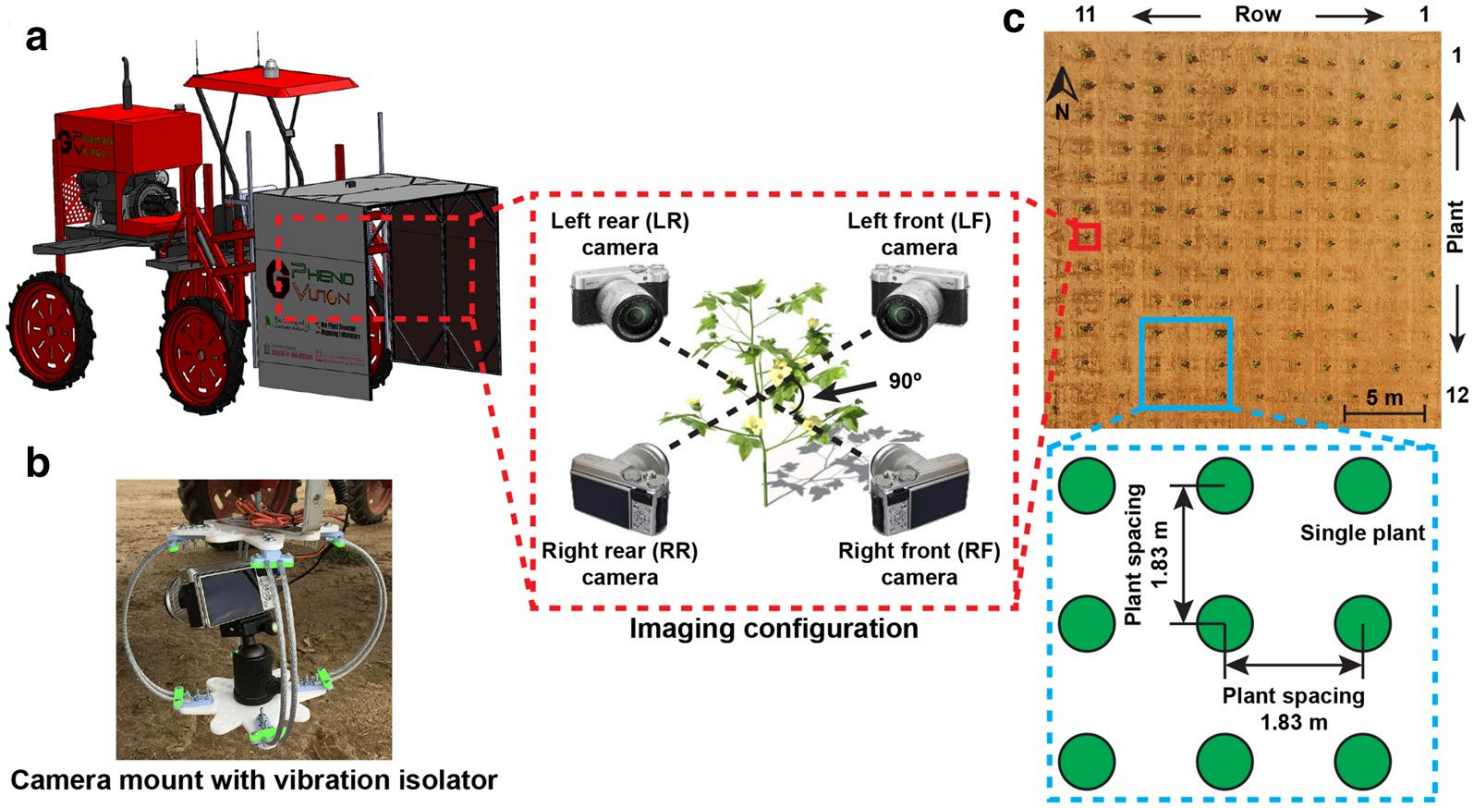
Diagram of the data acquisition system and field layout. **a** GPhenoVision system with the color imaging module for acquiring four-view images of plants. **b** Implementation of a specially designed camera mount for isolating high frequency vibration. **c** The single plant layout (SPL) field used in the present study

A previously developed ground mobile imaging system (“GPhenoVision” by Jiang et al. [22]) was modified with a multi-view color imaging module for data acquisition (Fig. 8a). The multi-view color imaging module consisted of four consumer grade mirror-less cameras (X-A10, Fujifilm Holdings Corporation, Tokyo, Japan) that faced towards the center of the system enclosure approximately 90° apart from neighboring cameras. To avoid potential issues of image quality (e.g., blurry images) because of high-frequency vibration, an inexpensive camera mount was manufactured by combining a camera ball mount and a vibration isolator, providing the flexibility of viewing angle configuration and the capability of isolating high-frequency vibrations (Fig. 8b). A custom trigger device was developed to synchronize triggering signals to all four cameras. The trigger device and an RTK-GPS (Cruizer II, Raven Industries Inc., Sioux Falls, SD, USA) were connected to a laptop computer in which a custom LabVIEW program ran to automatically save timestamps of triggering signals and RTK-GPS records. The developed data acquisition system acquired four color images at a time with RTK-GPS information.

Cotton seeds of 24 genotypes (from 3 genetic categories including *Gossypium hirsutum*, *Gossypium hirsutum*, and *Gossypium barbadense*) were planted in pots in a greenhouse on 13 June 2018 to obtain cotton seedlings. An experimental field was transplanted with 132 cotton seedlings (12 plants per row × 11 rows) in a SPL where individual plants (treated as one plot) had an in-row and across-row width of 1.52 m (Fig. 8c). Two batches of transplanting were conducted. The first batch of transplanting was conducted on 26 June 2018 (13 days after planting, DAPs), yielding 75 (out of 89 survived seedlings) healthy plants over the growing season. The second batch of transplanting was conducted on 5 July 2018 (22 DAPs), yielding additional 41 (out of 43 survived seedlings) healthy plants. A total of 116 plants from 23 genotypes, therefore, were used in the present study. The modified GPhenoVision system imaged the field in a continuous scanning mode every 2 days (or 3 days if over weekends) during the flowering period from 20 August 2018 (68 DAPs) to 24 October 2018 (133 DAPs).

### DeepFlower for characterization of flowering patterns

#### Image preprocessing and labeling

Collected images were segregated to individual plants based on the collection location information, generating a dataset containing 8666 images collected for 116 plants on 26 dates. A total of 7 plants were randomly identified and all 475 images of those 7 plants were used for manual annotation with bounding boxes of five classes (see Additional file 1: Figure S1). The five classes included the target plant, emerging bloom, opened cotton boll, region with specular reflectance, and others (objects other than the four classes). This labeling strategy was named as the 5-class labeling strategy. The 475 images were randomly shuffled to form training (380 images) and testing (95 images) datasets for training and evaluating object detection models. It should be noted that the 475 annotated images were exclusively used for training and validating the detection models. Analyses for the counting performance of the DeepFlower approach would not use the 475 images to avoid potential biases in the results. For the flowering pattern analyses, flowering curves of the 7 plants derived from the DeepFlower approach were still used to ensure adequate replicates in statistical analysis.

#### Bloom detection

**Fig. 9:**
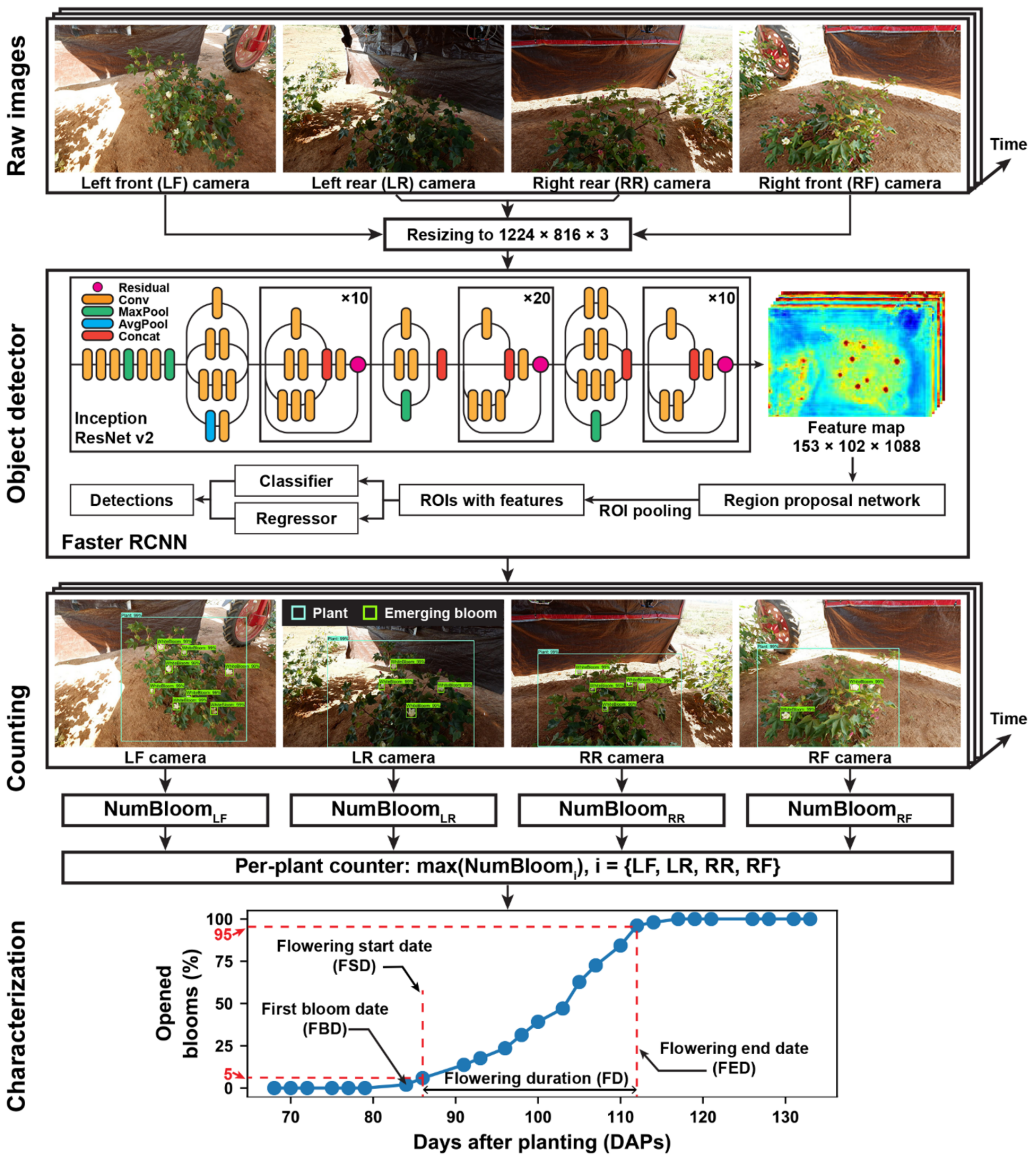
DeepFlower processing pipeline for detection, counting, and characterization of flowering patterns using deep learning method and color images

A deep learning-based approach (DeepFlower) was developed to detect and count emerging blooms in the collected images (Fig. 9). The approach consisted of three major sections including object detection, emerging bloom counting, and flowering characterization.

Object detection was the key of the DeepFlower approach. Because of the success of many object detection applications [23], the Faster RCNN model was used as the object detector in the present study (see Object detector in Fig. 9). The architecture contains a feature extractor, a region proposal network (RPN), and a classification and regressor module. The feature extractor is usually a deep CNN network, which extracts informative feature representations from the raw input images in a hierarchical fashion. The RPN uses the extracted features to generate potential regions of interest (ROIs), and the classification and regressor module uses the features in each ROI to identify the ROI class and refine the coordinates of ROI bounding box. As images contained diverse object classes with a similar appearance, the Inception ResNet v2 was used as a feature extractor due to its strong capability of learning adequate features to differentiate similar object classes.

As a limited number of labeled images were available, a transfer learning technique was used to facilitate model training. A Faster RCNN model was initialized using weights pretrained on the Common Objects in Context dataset (aka. COCO dataset, a large annotated image dataset open to the public) and fine-tuned on the training dataset for bloom detection. As the Faster RCNN model was trained using images labeled by the 5-class labeling strategy, the model was named as FrRCNN_5-cls_ for conciseness. Model training was performed using a mini-batch stochastic gradient descent (SGD, batch size was 2) by the Adam optimizer with an initial learning rate of 5 × 10^–5^, a dropout rate of 0.5 for the RPN and classification and regressor modules, and a weight decay of 1 × 10^–3^. Based on preliminary experiments, a total 50,000 training steps (equivalent to 167 epochs) were used to ensure the model convergence for the bloom detection task. Model checkpoints were saved after every 5,000 training iterations. Checkpoints with the best validation performance were selected for successive bloom counting analyses, which mimics the early stopping strategy to avoid potential overfitting issues. Two computing nodes (14 2.8 GHz CPU cores, 120 GB RAM, and Tesla V100 16 GB GPU memory) hosted by the Georgia Advanced Computing Resource Center (GACRC) were used for model training under the operating system of CentOS 7.5 with Tensorflow 1.12.0.

The trained Faster RCNN model could detect up to 100 bounding boxes of target plant and emerging blooms with classification confidence scores in a given image. If the confidence score was less than an arbitrary threshold (0.7 in the present study), a detection was removed from the detection result. Consequently, the final detection results contained only detections with a high classification confidence, which were used for bloom counting.

#### Bloom counting

A counting strategy was developed to use detection results from the Faster RCNN model to count the number of emerging blooms for a plant on one day (see Counting in Fig. 9). The strategy counted the number of emerging blooms for a plant in two steps. In the first step, emerging bloom detections were treated as blooms within the target plant if the centroids of their bounding boxes were within the bounding box of the target plant detection. Subsequently, the number of emerging blooms on the target plant was obtained for each of the four images acquired for a plant on one day. This provided an accurate bloom count for a plant from each of the four viewing angles. In the second step, we hypothesized that most (or all) emerging blooms should be seen from one of the four viewing angles, and thus the strategy selected the image (viewing angle) that provided the maximum bloom count from the four images as the number of emerging blooms for a plant on that day. Based on preliminary experiments, the maximum bloom count substantially outperformed the total bloom count and the average bloom count from the four images of a plant. As the first step only considered emerging blooms within a target plant, this counting strategy was summarized as the “plant-based counting” strategy.

#### Flowering characterization

The numbers of emerging blooms per plant per day over the flowering period were used to derive flowering curves for individual plants (see Characterization in Fig. 9). A flowering curve was defined as the cumulative percentage of opened blooms over the growing time (in DAPs). Cumulative percentage of opened blooms on individual days was calculated using Eq. 1.

1 $${P}_{k}=\frac{\sum_{i=0}^{k}{C}_{i}}{\sum_{i=0}^{N}{C}_{i}}$$

where *P*_*k*_ is the cumulative percentage of opened blooms for a plant on the *k*th DAPs, *C*_*i*_ is the count of emerging blooms for that plant on the *i*th DAPs, and N is the end day of the flowering period.

Three critical points were defined on a flowering curve, including first bloom date (FBD) when the first bloom was identified, flowering start date (FSD) when at least 5% of emerging blooms occurred on a plant, and flowering end date (FED) when at least 95% of emerging blooms occurred on a plant. Three flowering characteristics were derived from the three critical points. FBD and FSD were directly used as flowering characteristics, whereas FSD and FED were used to calculate flowering duration (FD), which was important for many applications related to improvements of environment adaptability.

### Ablation experiments

#### Labeling strategy

While image labeling seems straightforward, it could significantly affect the performance of trained deep neural networks. For the bloom detection task, a simple class definition was used to label images for training, including only three classes i.e., target plant, emerging bloom, and non-bloom. The non-bloom class contained all regions that were labeled other than plant and emerging bloom classes. This labeling strategy has been mostly used by many deep learning applications, which annotated only objects of interest. For brevity, this labeling strategy was named as the 3-class labeling strategy. Accordingly, the same training process was applied to train another Faster RCNN model (FrRCNN_3-cls_) using images labeled by the 3-class labeling strategy. This model was compared with the FrRCNN_5-cls_ model in terms of detection accuracy.

#### Counting strategy

The “plant-based counting” strategy would provide an accurate count of emerging blooms on a target plant in an image, but it required additional efforts on labeling (e.g., annotating target plants in images) and computing (e.g., judgement of emerging bloom location within or outside of a target plant). A simplified counting strategy was to directly use the number of emerging bloom detections as the count for a plant in an image, which might save those labeling and computing efforts. This simplified strategy could be valid, because images were captured for a single plant and might not contain much information of neighboring plants. As this strategy would use all emerging bloom detections in an image, it was named as the “whole image-based counting” strategy. An ablation experiment was conducted to compare the two counting strategies in terms of counting accuracy.

### Statistical analysis

For detection and counting accuracies, simple linear regression analyses were performed between imaging derived and manual counts for the 116 plants on 26 dates. No interception term was used for those analyses. The slope of regression equation, coefficient of determination (R^2^), and root mean squared error (RMSE) were used as indicators to evaluate performance. In addition, error analyses were conducted for the optimal counting approach (the combination of the best detection model and counting strategy) for both absolute counting and cumulative percentage calculation.

For flowering characteristics, analysis of variance (ANOVA) analyses were performed on the three flowering characteristics (FBD, FSD, and FD) among three genetic categories and genotypes, respectively, exploring differences in flowering patterns between various cultivated and exotic species. All tests were performed in R using a significance level of 0.05.

An important aspect of the present study is to guide the design of future large-scale experiments. The minimum replication number, therefore, was estimated for each flowering characteristic for experiments that are likely to include at least 200 genotypes from one population in a nested association mapping (NAM) study for cotton. Estimation was performed using the one-way ANOVA model with an effect size calculated using the present study data, a significance level of 0.05, and a statistical power of 0.95 in the G*Power software [24].

### Data collection frequency

Data collection frequency is an important factor in studies related to plant flowering patterns because it determines the temporal resolution of data and the cost of data acquisition. An optimal frequency would provide adequate information to discern flowering patterns among groups and reduce investments in data collection and management. To investigate this factor, the data collection frequency of the original dataset was reduced from three times per week (approximately 2–3 days between two sampling dates) to twice per week (approximately 3–4 days) and once per week (7 days), which formed two subsets. The two subsets were analyzed using the optimal processing approach to derive cumulative flowering curves and flowering characteristics. Average differences were calculated between the cumulative flowering curves derived by the imaging method and manual method with different data collection frequencies, evaluating effects caused by the difference in data collection frequency. In addition, extracted flowering characteristics were used in statistical analyses to examine the statistical power of different data collection frequencies. Through these efforts, the optimal data collection frequency would be determined and can be used to guide data collection in future studies.

## Supplementary Information

**Additional file 1:** **Figure S1.** Examples of objects labeled using the 5-class labeling strategy. **Figure S2.** Absolute bloom counting curves generated using imaging-derived and manual counts for three genetic categories (elite *G. hirsutum*, exotic *G. hirsutum*, and *G. barbadense*) in both the first and second transplanting batches. **Figure S3.** Absolute bloom counting curves generated using imaging-derived and manual counts for 23 genotypes in the first transplanting batch. **Figure S4.** Cumulative flowering curves derived using the imaging and manual counts for 23 genotypes in the first transplanting batch. **Figure S5.** Boxplot of flowering characteristics (first bloom date, flowering start date, and flowering duration) among three genetic categories (elite *G. hirsutum*, exotic *G. hirsutum*, and *G. barbadense*) in the first transplanting batch. **Figure S6.** Boxplot of flowering characteristics (first bloom date, flowering start date, and flowering duration) among three genetic categories (elite *G. hirsutum*, exotic *G. hirsutum*, and *G. barbadense*) in the first transplanting batch. **Table S1.** Overall ANOVA Table for first bloom date (FBD) calculated using manual counts. **Table S2.** Overall ANOVA Table for flowering start date (FSD) calculated using manual counts. **Table S3.** Overall ANOVA Table for flowering duration (FD) calculated using manual counts. **Table S4.** Overall ANOVA Table for first bloom date (FBD) calculated using imaging counts. **Table S5.** Overall ANOVA Table for flowering start date (FSD) calculated using imaging counts. **Table S6.** Overall ANOVA Table for flowering duration (FD) calculated using imaging counts.

## Data Availability

Calculated flowering curves and extracted flowering characteristics were associated in the supplementary csv files: flower_curves.csv and flower_characteristics.csv.
