# Supplementary material for: DeepFlower: a deep learning-based approach to characterize flowering patterns of cotton plants in the field
Source: Plant Methods. 2020 Dec 7;16:156. doi: 10.1186/s13007-020-00698-y (PMC7720604; doi:10.1186/s13007-020-00698-y)
Supplement: Supplementary file 1 — Additional file 1: Figure S1. Examples of objects labeled using the 5-class labeling strategy. Figure S2. Absolute bloom counting curves generated using imaging-derived and manual counts for three genetic categories (elite G. hirsutum, exotic G. hirsutum, and G. barbadense) in both the first and second transplanting batches. Figure S3. Absolute bloom counting curves generated using imaging-derived and manual counts for 23 genotypes in the first transplanting batch. Figure S4. Cumulative flowering curves derived using the imaging and manual counts for 23 genotypes in the first transplanting batch. Figure S5. Boxplot of flowering characteristics (first bloom date, flowering start date, and flowering duration) among three genetic categories (elite G. hirsutum, exotic G. hirsutum, and G. barbadense) in the first transplanting batch. Figure S6. Boxplot of flowering characteristics (first bloom date, flowering start date, and flowering duration) among three genetic categories (elite G. hirsutum, exotic G. hirsutum, and G. barbadense) in the first transplanting batch. Table S1. Overall ANOVA Table for first bloom date (FBD) calculated using manual counts. Table S2. Overall ANOVA Table for flowering start date (FSD) calculated using manual counts. Table S3. Overall ANOVA Table for flowering duration (FD) calculated using manual counts. Table S4. Overall ANOVA Table for first bloom date (FBD) calculated using imaging counts. Table S5. Overall ANOVA Table for flowering start date (FSD) calculated using imaging counts. Table S6. Overall ANOVA Table for flowering duration (FD) calculated using imaging counts. [file 13007_2020_698_MOESM1_ESM.docx]

**DeepFlower: A deep learning-based approach to characterize flowering patterns of cotton plants in the field**

**Supplementary materials**

Yu Jiang^1,2^, Changying Li^2,*^^[[1]](#footnote-1)^, Rui Xu^2^, Shangpeng Sun^2^, Jon S. Robertson^3^, and Andrew H. Paterson^3,4^

^1^Horticulture Section, School of Integrative Plant Science, Cornell AgriTech, Cornell University, Geneva, NY 14456

^2^School of Electrical and Computer Engineering, College of Engineering, The University of Georgia, Athens, GA 30602

^3^College of Agricultural & Environmental Sciences, The University of Georgia, Athens, GA 30602

^4^Franklin College of Arts and Sciences, The University of Georgia, Athens, GA 30602


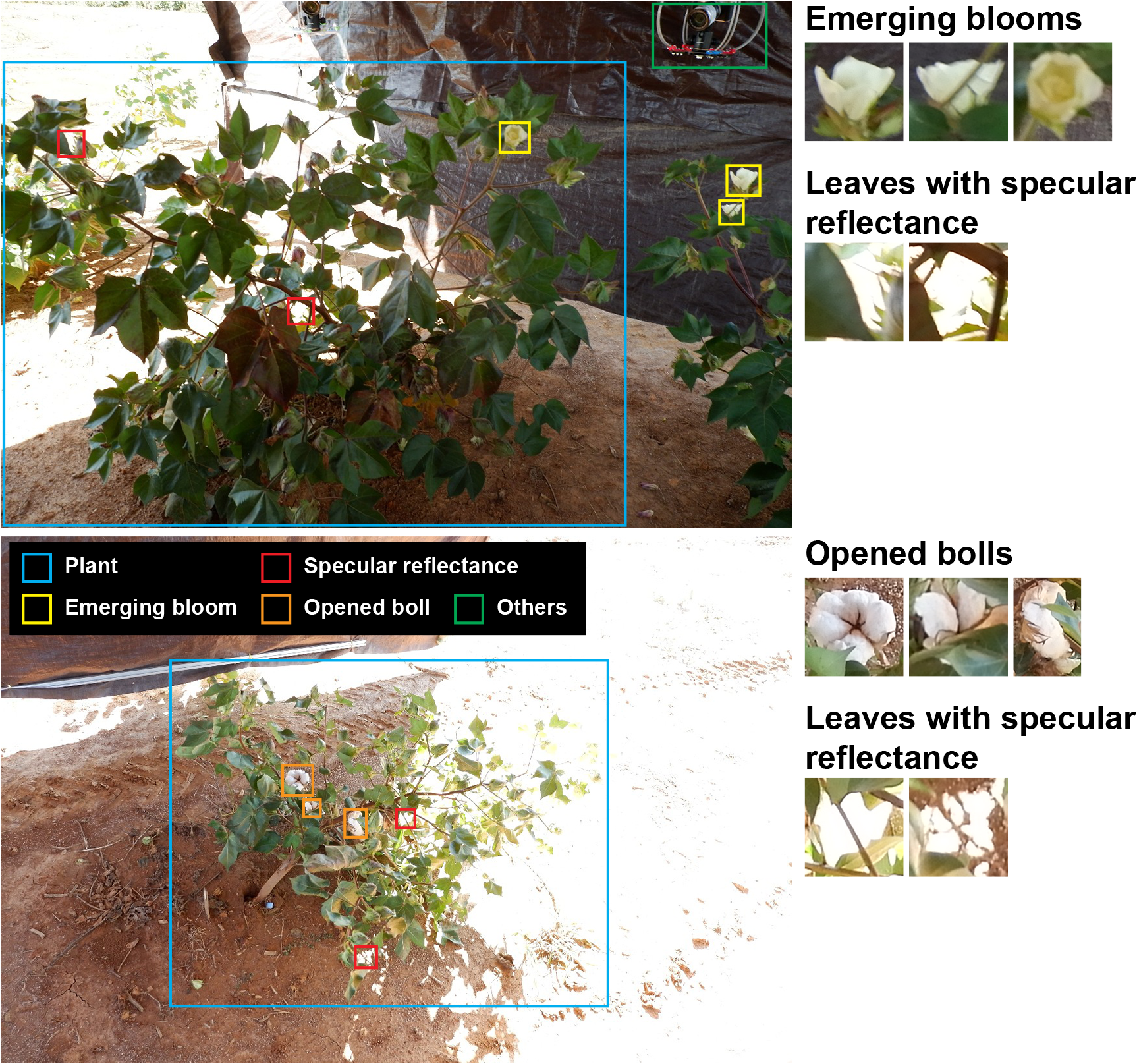


**Figure S1.** Examples of objects labeled using the 5-class labeling strategy. For the 3-class labeling strategy, classes other than “Plant” and “Emerging bloom” were merged into one class “Others”.


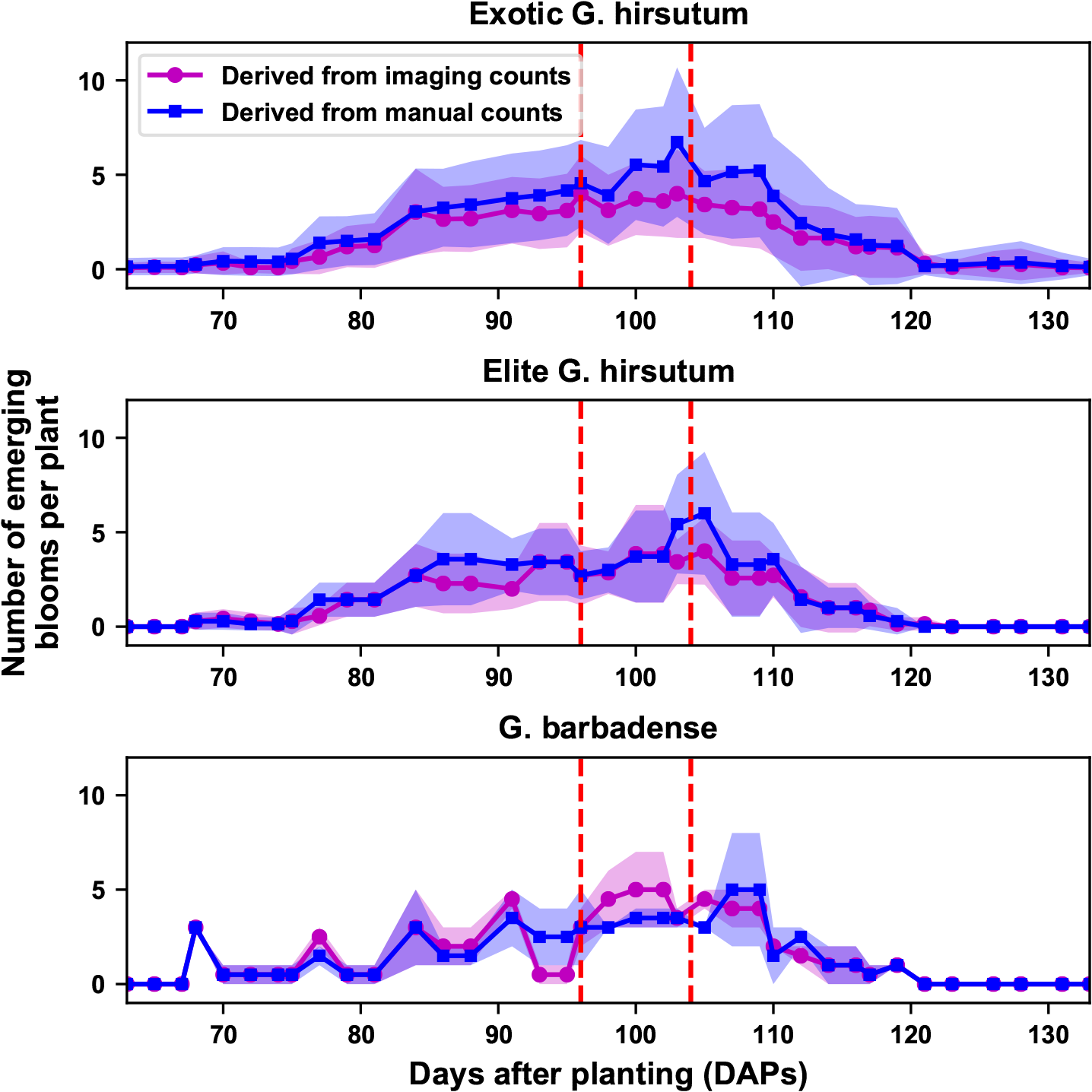


**Figure S2.** Absolute bloom counting curves generated using imaging-derived and manual counts for three genetic categories (elite *G. hirsutum*, exotic *G. hirsutum*, and *G. barbadense*) in both the first and second transplanting batches. Group mean values were drawn in solid lines and group standard deviations were indicated by shaded areas (magenta and blue for curves generated using imaging-derived and manual counts, respectively). The two red dashed lines indicated two days when the field received a heavy rain and chilling temperature: the left line indicated 16 September 2018 (95 DAPs) and the right line indicated 24 September 2018 (103 DAPs).


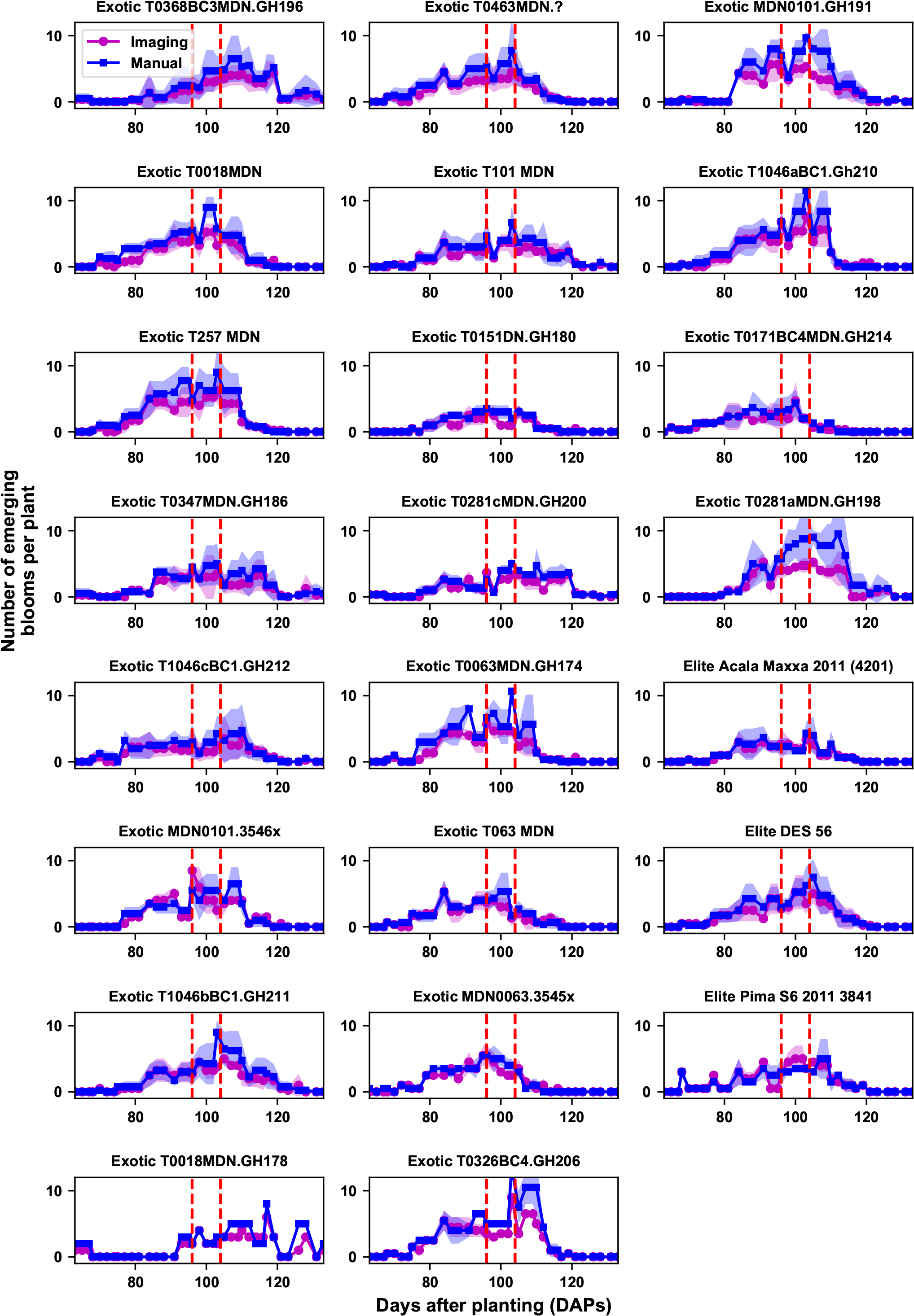


**Figure S3.** Absolute bloom counting curves generated using imaging-derived and manual counts for 23 genotypes in the first transplanting batch. Group mean values were drawn in solid lines and group standard deviations were indicated by shaded areas (magenta and blue for curves generated using imaging-derived and manual counts, respectively). The two red dashed lines indicated two days when the field received a heavy rain and chilling temperature: the left line indicated 16 September 2018 (95 DAPs) and the right line indicated 24 September 2018 (103 DAPs).


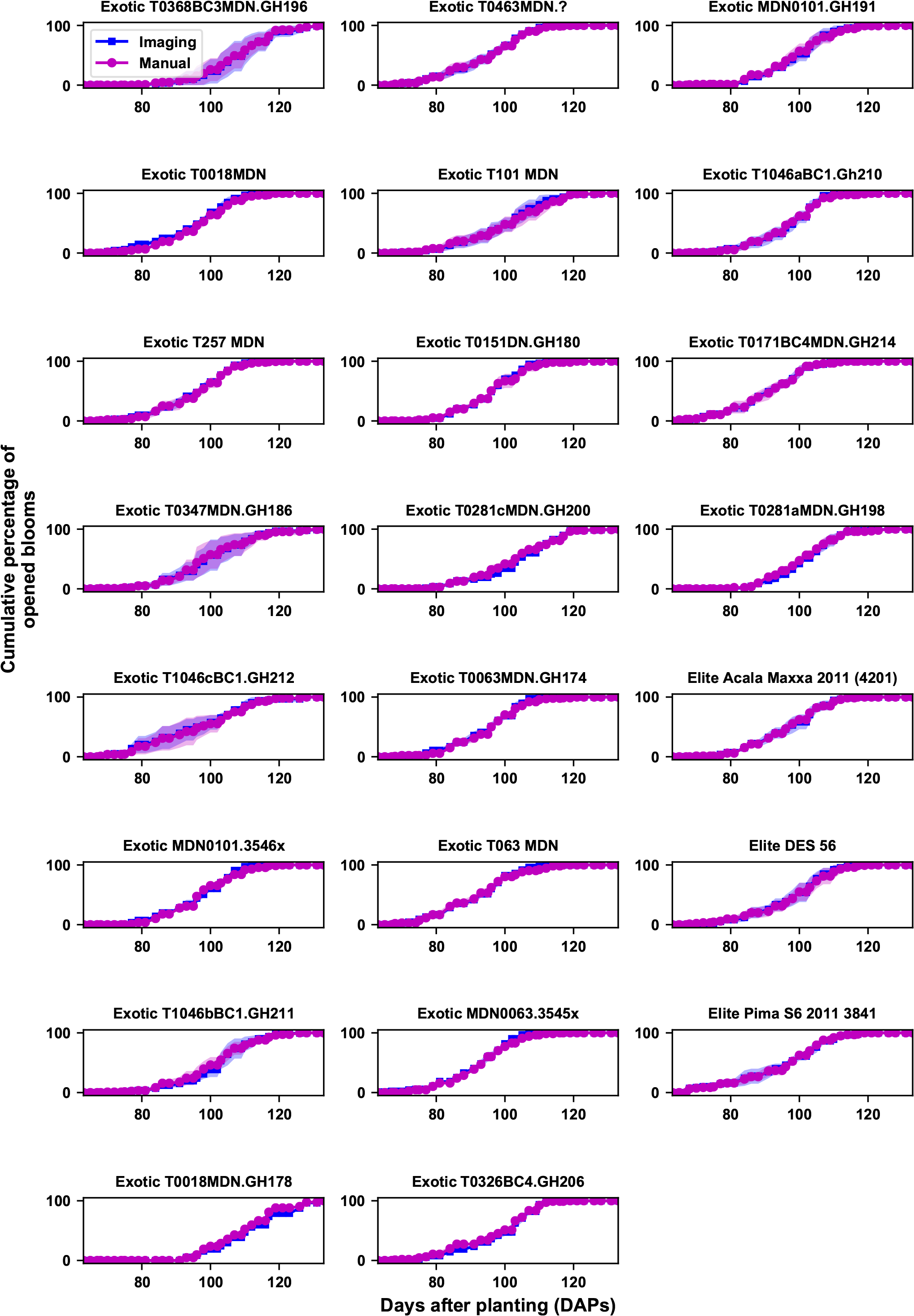


**Figure S4.** Cumulative flowering curves derived using the imaging and manual counts for 23 genotypes in the first transplanting batch. Group mean values were drawn in solid lines and group standard deviations were indicated by shaded areas (magenta for imaging-count derived results and blue for manual-count derived results).


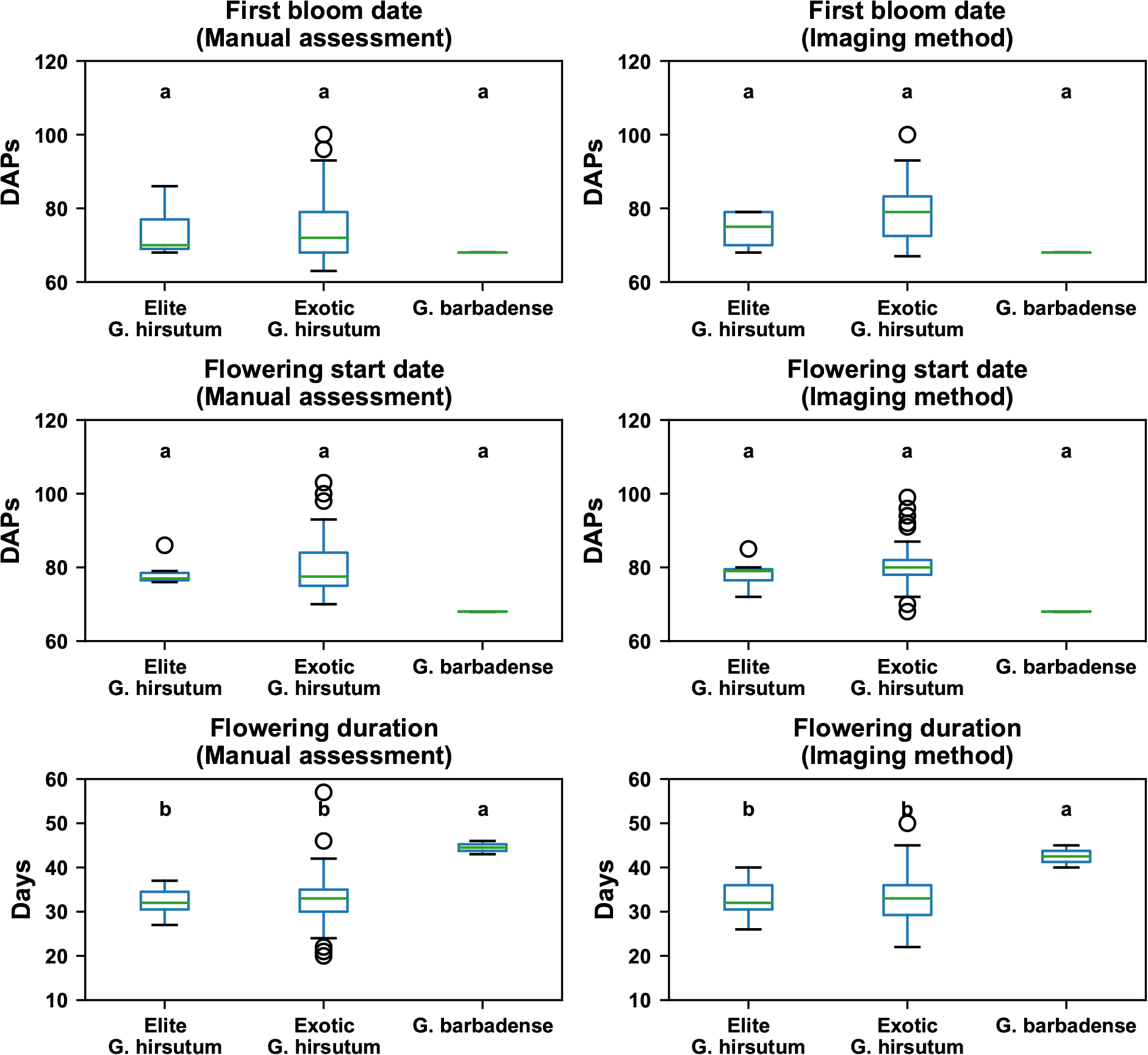


**Figure S5.** Boxplot of flowering characteristics (first bloom date, flowering start date, and flowering duration) among three genetic categories (elite *G. hirsutum*, exotic *G. hirsutum*, and *G. barbadense*) in the first transplanting batch. Groups with a statistically significant difference (p < 0.05) are denoted with different letters, and group mean values of each characteristic are sorted alphabetically. The flowering characteristics were calculated using the subset with a data collection frequency of twice per week.


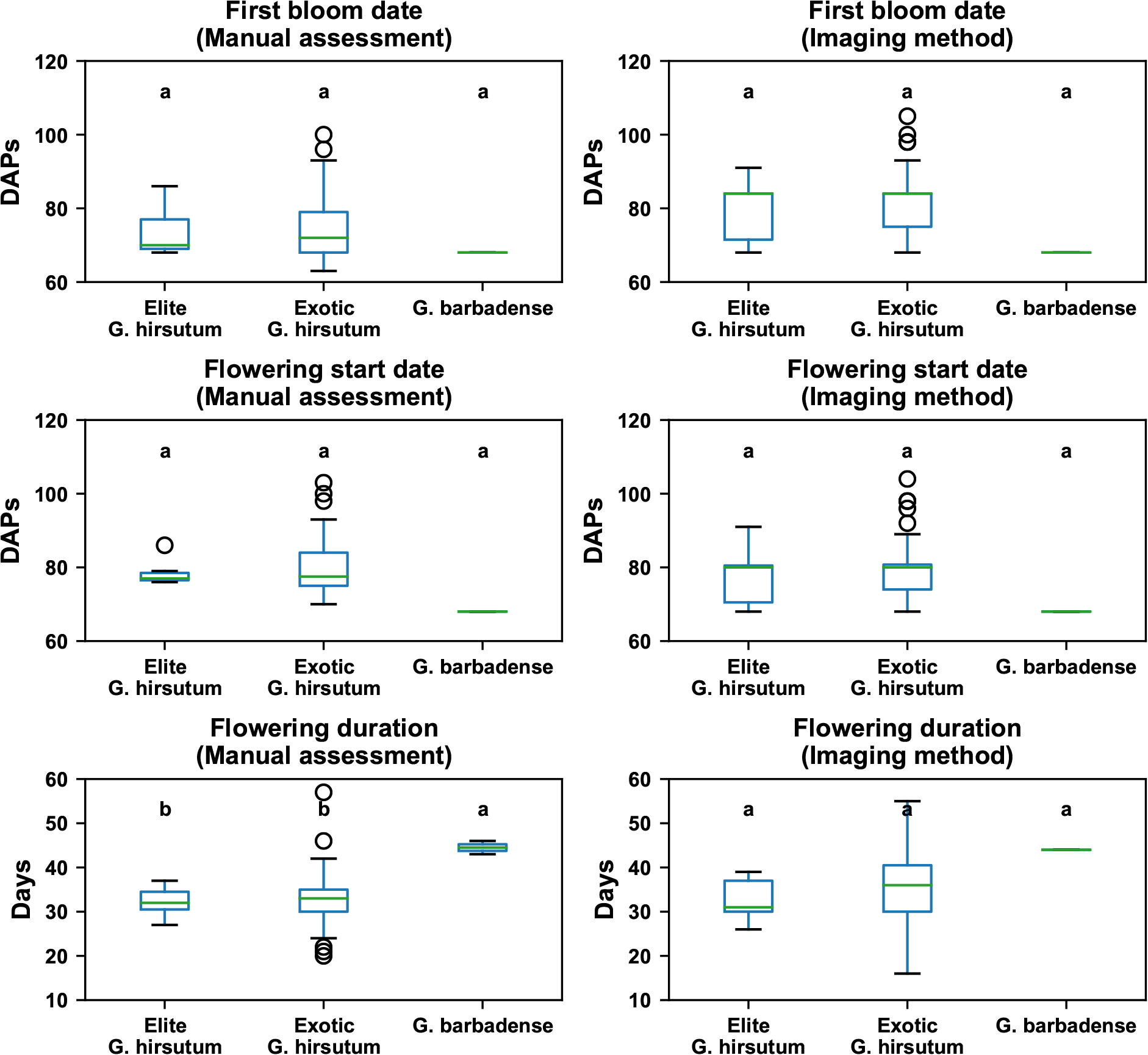


**Figure S6.** Boxplot of flowering characteristics (first bloom date, flowering start date, and flowering duration) among three genetic categories (elite *G. hirsutum*, exotic *G. hirsutum*, and *G. barbadense*) in the first transplanting batch. Groups with a statistically significant difference (p < 0.05) are denoted with different letters, and group mean values of each characteristic are sorted alphabetically. The flowering characteristics were calculated using the subset with a data collection frequency of once per week.

Interaction effects between genotype and transplanting date consistently showed statistical significance in ANOVA analyses for all flowering characteristics, so it is necessary to separate samples from different transplanting batches for analyzing the genotype effect exclusively.

**Table S1.** Overall ANOVA Table for first bloom date (FBD) calculated using manual counts

| Effect | Degree of freedom | Sum of squares | Mean sum of squares | F-value | P-value |
| --- | --- | --- | --- | --- | --- |
| Genotype | 22 | 4412 | 200.53 | 6.379 | <0.001*** |
| Transplanting date | 1 | 113 | 113.05 | 3.596 | 0.062 |
| Interaction | 19 | 1120 | 58.97 | 1.876 | 0.029* |
| Residual | 73 | 2295 | 31.44 |  |  |

**Table S2.** Overall ANOVA Table for flowering start date (FSD) calculated using manual counts

| Effect | Degree of freedom | Sum of squares | Mean sum of squares | F-value | P-value |
| --- | --- | --- | --- | --- | --- |
| Genotype | 22 | 3607 | 163.97 | 8.926 | <0.001*** |
| Transplanting date | 1 | 137 | 137.04 | 7.460 | 0.008** |
| Interaction | 19 | 707 | 37.20 | 2.025 | 0.017* |
| Residual | 73 | 1341 | 18.37 |  |  |

**Table S3.** Overall ANOVA Table for flowering duration (FD) calculated using manual counts

| Effect | Degree of freedom | Sum of squares | Mean sum of squares | F-value | P-value |
| --- | --- | --- | --- | --- | --- |
| Genotype | 22 | 1265.1 | 57.50 | 2.179 | 0.007*** |
| Transplanting date | 1 | 49 | 49.04 | 1.858 | 0.177 |
| Interaction | 19 | 966.1 | 50.85 | 1.927 | 0.024* |
| Residual | 73 | 1926.3 | 26.39 |  |  |

**Table S4.** Overall ANOVA Table for first bloom date (FBD) calculated using imaging counts

| Effect | Degree of freedom | Sum of squares | Mean sum of squares | F-value | P-value |
| --- | --- | --- | --- | --- | --- |
| Genotype | 22 | 3721 | 169.12 | 5.436 | <0.001*** |
| Transplanting date | 1 | 83 | 83.15 | 2.673 | 0.106 |
| Interaction | 19 | 1219 | 64.15 | 2.062 | 0.015* |
| Residual | 73 | 2271 | 31.11 |  |  |

**Table S5.** Overall ANOVA Table for flowering start date (FSD) calculated using imaging counts

| Effect | Degree of freedom | Sum of squares | Mean sum of squares | F-value | P-value |
| --- | --- | --- | --- | --- | --- |
| Genotype | 22 | 3164 | 143.83 | 8.879 | <0.001*** |
| Transplanting date | 1 | 106 | 106.26 | 6.560 | 0.013* |
| Interaction | 19 | 677 | 35.65 | 2.201 | 0.009** |
| Residual | 73 | 1182 | 16.20 |  |  |

**Table S6.** Overall ANOVA Table for flowering duration (FD) calculated using imaging counts

| Effect | Degree of freedom | Sum of squares | Mean sum of squares | F-value | P-value |
| --- | --- | --- | --- | --- | --- |
| Genotype | 22 | 1322.5 | 60.11 | 2.480 | 0.002** |
| Transplanting date | 1 | 93.8 | 93.79 | 3.869 | 0.053 |
| Interaction | 19 | 1187.8 | 62.52 | 2.579 | 0.002** |
| Residual | 73 | 1769.5 | 24.24 |  |  |

1. **Author contributions:** C.L., Y.J., and R.X. conceived the original concept; Y.J and A.H.P. designed the experiments; Y.J., R.X., and S.S. developed the data acquisition system; J.S.R. conducted the experiment and most field data collection; Y.J. developed the DeepFlower processing pipeline; Y.J., A.H.P., and C.L. analyzed the data; Y.J., C.L., and A.H.P. wrote the article with contributions of all the authors; C.L. agrees to serve as the author responsible for contact and ensures communication.

   **Responsibilities of the Author for Contact:** Changying Li is the author for contact and ensures that all scientists who have contributed substantially to the conception, design or execution of the work described in the manuscript are included as authors, and that all authors agree to the list of authors and the identified contributions of those authors.

   **Funding information:** This study was funded by the National Robotics Initiative (NIFA grant No: 2017-67021-25928). This project is also supported by Cotton Incorporated (17-510GA).

   ***Correspondence to:** Changying Li (cyli@uga.edu) at 712F Boyd Graduate Studies, University of Georgia, Athens, GA 30602. [↑](#footnote-ref-1)
